# Clinical Predictors of Adherence to Low Tidal Volume Ventilation Practice: Is it Different on Weekend and Night Shifts?

**DOI:** 10.7759/cureus.4844

**Published:** 2019-06-06

**Authors:** Rashid N Nadeem, Ashraf M Elhoufi, Mohamed A Soliman, Islam Bon, Zaineb A Obaida, Mayada M Hussien, Lamiaa Salama, Ahmed N Elsousi, Sahish Kamat, Rami M Satti, Naheed Elahi, Raed H Abuhijleh, Moatz G ElZeiny, Hitham Fargaly, Mohamed M Ibrahim

**Affiliations:** 1 Intensive Care Medicine, Dubai Hospital, Dubai, ARE

**Keywords:** low tidal volume ventilation, hypoxemia, lung protective ventilation, acute lung injury, adherence, hypercapnia, outcomes, low tidal volume ventilation, shifts

## Abstract

Background: Low tidal volume ventilation (LTVV) strategy improves outcomes; however, despite recommended by guidelines, adherence to this practice is not high.

Methods: Tidal volume for mechanically ventilated patients were recorded for each 12-hour shift, day and night shifts for consecutive 101 patients. Adherence was determined by comparing these tidal volumes to standard low tidal volumes of 6 ml/kg of ideal body weight. Adherence rates were calculated and adherence rates of daytime shifts were compared to those of night time shifts. Adherence rates for weekday shifts were compared with those of weekend shifts. Clinical variables were recorded to analyze predictors of adherence pattern.

Results: The sample size was 101 patients with 870 patient-ventilator days with 1734 patient ventilator shifts. Shift adherence was only 47.5%. There was no significant difference between day and night shifts or weekday and weekend shifts. Stepwise multiple regression analysis shows that age, gender, body mass index (BMI), and partial pressure of carbon dioxide (PCO2) have significant correlation with adherence to LTVV practice.

Conclusion: The study found that adherence to lung protective low tidal volume mechanical ventilation practice is low. Practice adherence is not different over weekend or night shifts. Age, gender, BMI, and PCO2 have significant correlation with adherence to LTVV practice.

## Introduction

Respiratory failure is a syndrome rather than a single disease process. The overall frequency of respiratory failure is not well known [1], for which patients require mechanical ventilation. Positive pressure ventilation is non physiologic and may cause adverse effects including pulmonary barotrauma and its associated negative consequences on the lung. It may lead to alveolar rupture resulting in the release of air into extra-alveolar spaces [2]. The incidence of barotrauma during mechanical ventilation varies with the underlying indication for mechanical ventilation but ranges from 0%-50% [3]. Low tidal volume ventilation (LTVV) is referred to as lung protective ventilation that aims to avoid alveolar over-distension, which is one of the principal mechanisms of ventilator-induced lung injury (VILI). VILI may lead to further pulmonary damage by causing increasing alveolar permeability, interstitial and alveolar edema, alveolar hemorrhage, hyaline membranes, loss of functional surfactant, and alveolar collapse [4-7]. LTVV is recommended to be 4 mL/kg to 8 mL/kg of predicted ideal body weight provided that inspiratory plateau airway pressure remains ≤30 cm H2O [8]. Collectively, evidence suggests that the early application of and adherence to LTVV decreases mortality as well as other clinically important outcomes in patients with acute respiratory distress syndrome (ARDS) [9]. Although proved beneficial, LTVV has negative effects including increased need for sedation, auto-PEEP (positive end expiratory pressure) from resultant high frequency of respiratory rate to achieve target minute ventilation, which may cause hypercapnic respiratory acidosis [10-11]. There are conditions in which this high partial pressure of carbon dioxide (PCO2) has deleterious consequences such as acute cerebral disease, coronary artery disease, heart failure, cardiac arrhythmias, pulmonary hypertension with right ventricular dysfunction plus hypovolemia; so these conditions are considered to be relative contra-indications of LTVV strategy [12-13].

In two meta-analyses including 15 and seven randomized trials, respectively, LTVV strategy in non-ARDS ventilated medical and surgical patients was associated with lower mortality, less incidence of ventilator associated pneumonia (VAP) plus ventilator associated lung injury (VALI) in addition to less progression to ARDS [14-15]. Despite robust evidence demonstrating multiple benefits of LTVV in ARDS, the acceptance of this practice is disturbingly low. Major barriers to utilization of LTVV are the inability to recognize and tailor tidal volume to ideal body weight (IBW) (a function of height and gender) and use of non-volume control modes of ventilation. Less common barriers to the implementation of LTVV include the fear of increased sedation needs, lack of acceptance of permissive hypercapnia, management of refractory hypoxemia, and multidisciplinary team dynamics [16-17]. Finally, the most important barrier is poor recognition of ARDS by clinicians [18]. We conducted a retrospective medical records review study to determine adherence to this standard practice of LTVV at our community hospital and to investigate if there is a different pattern in adherence practice over weekend days in comparison to weekdays. We also aimed to determine if there are any clinical factors that are correlated with adherence.

## Materials and methods

We performed a retrospective medical records review study at a single medical center, Dubai Hospital, Dubai. All medical records of mechanically ventilated patients who were admitted to the intensive care unit of the Dubai Hospital from August 2017 to July 2018 were reviewed if they met the following inclusion criteria.

Inclusion criteria

All patients admitted to the ICU who received mechanical ventilation for more than 24 hours.

Exclusion criteria

The exclusion criteria were contraindication to LTVV (intracranial pathology, intracranial hemorrhage (ICH), cerebrovascular accident (CVA), known high previous PCO2, acute exacerbation of chronic obstructive pulmonary disease (COPD) with high PCO2, cardiogenic shock).

Definitions

All patients’ ventilator settings (tidal volume (TV), positive end expiratory pressure (PEEP), and plateau pressure) were recorded daily from the morning shift (9AM-9PM) and at night (9PM-9AM).

Ideal body weight was calculated for each patient using the J. Devine Formula (1974) [19].

Male patients: IBW (kg) = 50 + 2.3 (height [inches] -60).

Female patients: IBW (kg) = 45.5 + 2.3 (height [inches] -60).

Male patients: IBW (kg) = 50 + 2.3 (height [cm]/2.54 -60).

Female patients: IBW (kg) = 45.5 + 2.3 (height [cm]/2.54 -60).

Tidal volume was calculated for all patients as 6 ml/kg of IBW.

Patient ventilator days were calculated by multiplying patient number by days of ICU stay with mechanical ventilation. Adherence was determined for each shift (day and night) by comparing ideal tidal volume and actual tidal volume.

Confounding variables affecting target measures

We also recorded the following variables that may affect adherence to this practice: primary diagnosis to classify ARDS versus non-ARDS diagnosis, tracheostomy versus endotracheal tube, X-ray chest abnormality (presence or absence of infiltrates), arterial blood gas PH, PCO2, partial pressure of oxygen (PaO2) and fraction of inspired oxygen (FiO2). Demographics (age, gender, BMI) were also recorded. We also recorded acute physiology and chronic health evaluation (APACHE) scores at 24 hours after admission to the ICU and the final outcome of the patient at discharge from the ICU (alive or dead).

Statistical analysis

Adherence rate was determined for day and night shifts as well as weekend and weekday shifts. These rates were compared. There was no significant difference in variables compared between adherent and non-adherent subjects. We conducted stepwise multiple regression analysis using the Statistical Package for the Social Sciences (SPSS) software Version 25.0 (IBM Corp. Armonk, NY).

## Results

Tidal volume for mechanically ventilated patients were recorded for each 12-hour shift; day and night for consecutive 101 patients. Adherence was determined by comparing these TVs to standard low TVs of 6 ml/kg of ideal body weight and adherence rates were calculated. Day shift tidal volume adherence was 459/870 (52.8%) versus night shift adherence 452/864 (52.3 %), p=0.44. Weekday adherence was 646/1263 (51.1%) versus weekends 265/471 (56.3%) p=0.05. Sample characteristics included age (59 +/- 20) years, gender M/F (61/40), BMI (27.1 +/- 8.1) kg/m2, APACHE II score (24.6 +/- 8.3), PEEP (7.12 +/- 2.3), FIO2 (62.9% +/- 21.4%), mortality (died 47/alive 48), endotracheal tube (ETT) versus tracheostomy (trach) (87/5), abnormal/normal chest X-ray (64/31), PH (7.27 +/- .14), PCO2 (43.07 +/- 18.8) torr and PO2 (134.9 +/- 106) torr. Stepwise multiple regression analysis showed that age, gender, BMI, and PCO2 have significant correlation with adherence to LTVV practice (Table 1). Younger patients, male patients, patients with less BMI and less PCO2 were more likely to have higher adherence rates.

**Table 1:** Stepwise multiple regression analysis (adherence against variables). APACHE - acute physiology and chronic health evaluation, BMI - body mass index, PEEP - positive end expiratory pressure, FiO2 - fraction of inspired oxygen, Trach/ETT - tracheostomy/endotracheal tube, CXR - chest x-ray, PO2 - partial pressure of oxygen, PCO2 - partial pressure of carbon dioxide

Variable	Mean	SD	N	Correlation	P value
Adherence %	51.8		101	1	
APACHE score	24.69	8.31	101	-0.022	0.413
Age (years)	59.03	20.01	101	-0.273	0.003
Gender M/F	61/40		101	-0.475	0.001
BMI (Kg/m2)	27.18	8.10	98	-0.198	0.025
PEEP (mm of water)	7.12	2.34	99	0.127	0.105
Plateau pressure (cm of water)	24.80	5.37	97	0.09	0.189
FiO2 (mm of Hg)	62.91	21.46	98	0.109	0.142
Dead/alive (N)	47/48		95	0.136	0.094
Trach/ETT (N)	5/87		92	-0.081	0.22
CXR infiltrate (present/Absent)	31/64		95	-0.012	0.453
pH	7.26	0.14	97	-0.055	0.297
PO2	134.94	106.13	97	0.117	0.127
PCO2	43.07	18.85	97	-0.228	0.013

## Discussion

Our study reveals that adherence to lung protective mechanical ventilation by employing low tidal volume ventilation is very poor-only about 50%-and there is no difference over weekends and night shifts. Studies showed low tidal volume ventilation strategy decreased mortality and increased the number of days without ventilator use in patients with acute lung injury and acute respiratory distress syndrome (ARDS) [20]. LTVV often requires higher level of positive end expiratory pressure (PEEP), and high levels of PEEP may improve survival in ARDS patients [21]. The practice benefit expands to non-ARDS patients as it provides similar benefit. Data regarding its generalized application in non-ARDS patients is not as significant as ARDS. On the contrary, use of LTVV intraoperatively with minimal PEEP is associated with an increased risk of 30-day mortality [22]. A more recent study showed that in patients without ARDS, low tidal volume strategy did not result in a greater number of ventilator-free days than an intermediate tidal volume strategy [23]. Many hospitals have standard protocols to ensure LTVV. Protocol directed interventions effectively decreased large tidal volumes and it was associated with a decreased frequency of new acute lung injury [24]. Protocols require an interdisciplinary approach and high number of healthcare staff, which is uncommon outside large centers, especially outside USA. Inadequate staffing (physicians and respiratory therapists) often plays a major role in adherence to standard protocols. Lower adherence in general in our study may be reflective of this factor.

Regarding patient-specific factors, we performed regression analysis, which showed that the APACHE score is not a predictor of adherence (coefficient -0.022, P=0.410). There was no significant difference for adherence for patients with endotracheal ventilation versus ventilated via tracheostomy. Mortality was also similar for both groups. Finding of x-ray lung infiltrate was not different between non-adherent and adherent groups. The only significant factors were age, gender, and high PCO2 levels on arterial blood gas, which is understandable, as patients with non-ARDS pathology who have high PCO2 may have lower adherence to LTVV prescription to maintain adequate ventilation. We believe these observations may not be significant as the sample size of our study was not large enough to conclusively disassociate these factors with adherence.

Our detailed shift to shift analysis showed that patients on non-adherent shifts have lower PEEP value, higher plateau pressure and higher peak airway pressure, which suggest that if these patients have LTVV, they may have higher PEEP, lower plateau pressure and peak pressure. It only emphasizes the fact that LTVV has beneficial mechanical effects on the lung.

We identified the following weaknesses of our study. It was a single center study with a relatively small sample size, so the results are not generalizable. It does reflect the true real life practice pattern though and highlights multiple areas for us to improve outcome. Retrospective chart review study provides only weak level of evidence, but it does identify that poor adherence is a significant issue. Data on adherence to such practices are not common in this part of the developing world. With the recent incorporation of electronic medical records, institutions are becoming better equipped to monitor and identify factors affecting practice patterns, which will improve the process of quality measurement, which will translate into improved clinical outcomes. We plan to implement protocols and study these quality measures in prospective patterns, which will help us determine predictors of good quality care and improved clinical outcomes.

## Conclusions

Adherence to low tidal volume mechanical ventilation practice appears to be about 50% only and it is not different over weekends or night shifts. Age, gender, BMI, and PCO2 have significant correlation with adherence to LTVV practice. Studies are needed, especially in developing countries, to explore and improve practice of low tidal volume mechanical ventilation.

## References

[1] Khan NA, Palepu A, Norena M (2008). Differences in hospital mortality among critically ill patients of Asian, Native Indian, and European descent. Chest.

[2] Doelken P, Sahn SA (2008). Pleural disease in the critically ill patient. Intensive Care Medicine, 6th Edition.

[3] Anzueto A, Frutos-Vivar F, Esteban A (2004). Incidence, risk factors and outcome of barotrauma in mechanically ventilated patients. Intensive Care Med.

[4] (1999). International consensus conferences in intensive care medicine: ventilator-associated lung injury in ARDS. Intensive Care Med.

[5] Slutsky AS, Ranieri VM (2013). Ventilator-induced lung injury. N Engl J Med.

[6] Rouby JJ, Brochard L (2007). Tidal recruitment and overinflation in acute respiratory distress syndrome: yin and yang. Am J Respir Crit Care Med.

[7] Hughes KT, Beasley MB (2017). Pulmonary manifestations of acute lung injury: more than just diffuse alveolar damage. Arch Pathol Lab Med.

[8] Fan E, Del Sorbo L, Goligher EC (2017). An Official American Thoracic Society/European Society of Intensive Care Medicine/Society of Critical Care Medicine Clinical Practice Guideline: mechanical ventilation in adult patients with acute respiratory distress syndrome. Am J Respir Crit Care Med.

[9] Acute Respiratory Distress Syndrome Network, Brower RG, Matthay MA (2000). Ventilation with lower tidal volumes as compared with traditional tidal volumes for acute lung injury and the acute respiratory distress syndrome. N Engl J Med.

[10] Hough CL, Kallet RH, Ranieri VM, Rubenfeld G, Luce J, Hudson L (2005). Intrinsic positive end-expiratory pressure in acute respiratory distress syndrome (ARDS) network subjects. Crit Care Med.

[11] Kregenow DA, Rubenfeld GD, Hudson LD, Swenson ER (2006). Hypercapnic acidosis and mortality in acute lung injury. Crit Care Med.

[12] Feihl F, Perret C (1994). Permissive hypercapnia. How permissive should we be?. Am J Respir Crit Care Med.

[13] Kavanagh BP, Laffey JG (2006). Hypercapnia: permissive and therapeutic. Minerva Anestesiol.

[14] Serpa Neto A, Cardoso SO, Manetta JA (2012). Association between use of lung-protective ventilation with lower tidal volumes and clinical outcomes among patients without acute respiratory distress syndrome: a meta-analysis. JAMA.

[15] Neto AS, Simonis FD, Barbas CS (2015). Lung-protective ventilation with low tidal volumes and the occurrence of pulmonary complications in patients without acute respiratory distress syndrome: a systematic review and individual patient data analysis. Crit Care Med.

[16] Needham DM, Yang T, Dinglas VD (2015). Timing of low tidal volume ventilation and intensive care unit mortality in acute respiratory distress syndrome. A prospective cohort study. Am J Respir Crit Care Med.

[17] Rubenfeld GD, Cooper C, Carter G, Thompson BT, Hudson LD (2004). Barriers to providing lung-protective ventilation to patients with acute lung injury. Crit Care Med.

[18] Weiss CH, Baker DW, Weiner S (2016). Low tidal volume ventilation use in acute respiratory distress syndrome. Crit Care Med.

[19] Devine BJ (1974). Gentamicin therapy. Drug Intell Clin Pharm.

[20] Acute Respiratory Distress Syndrome Network (2000). Ventilation with lower tidal volumes as compared with traditional tidal volumes for acute lung injury and the acute respiratory distress syndrome. New Engl J Med.

[21] Oba Y, Thameem DM, Zaza T (2009). High levels of PEEP may improve survival in acute respiratory distress syndrome: a meta-analysis. Respir Med.

[22] Levin MA, McCormick PJ, Lin HM, Hosseinian L, Fischer GW (2014). Low intraoperative tidal volume ventilation with minimal PEEP is associated with increased mortality. Br J Anaesth.

[23] Writing Group for the PReVENT Investigators (2018). Effect of a low vs intermediate tidal volume strategy on ventilator-free days in intensive care unit patients without ARDS: a randomized clinical trial. JAMA.

[24] Yilmaz M, Keegan MT, Iscimen R, Afessa B, Buck CF, Hubmayr RD, Gajic O (2007). Toward the prevention of acute lung injury: protocol-guided limitation of large tidal volume ventilation and inappropriate transfusion. Critical Care Med.

